# IDH Mutant Cholangiocarcinoma: Pathogenesis, Management, and Future Therapies

**DOI:** 10.3390/curroncol32010044

**Published:** 2025-01-17

**Authors:** Alexander Bray, Vaibhav Sahai

**Affiliations:** Division of Hematology and Oncology, Department of Internal Medicine, University of Michigan, Ann Arbor, MI 48109, USA; alebra@med.umich.edu

**Keywords:** cholangiocarcinoma, mutant isocitrate dehydrogenase, targeted therapy, ivosidenib, 2-hydroxyglutarate, R2HG

## Abstract

Mutations in isocitrate dehydrogenase (IDH) genes are among the most frequently encountered molecular alterations in cholangiocarcinoma (CCA). These neomorphic point mutations endow mutant IDH (mIDH) with the ability to generate an R-enantiomer of 2-hydroxyglutarate (R2HG), a metabolite that drives malignant transformation through aberrant epigenetic signaling. As a result, pharmacologic inhibition of mIDH has become an attractive therapeutic strategy in CCAs harboring this mutation. One such inhibitor, ivosidenib, has already undergone clinical validation and received FDA approval in this disease, but there is still much work to be done to improve outcomes in mIDH CCA patients. In this publication we will review the pathogenesis and treatment of mIDH CCA with special emphasis on novel agents and combinations currently under investigation.

## 1. Introduction

Cholangiocarcinoma (CCA) is a malignancy which arises from the mucosa of the biliary tree. These bile duct cancers are rare, with an incidence of less than 2.5 per 100,000 persons in the United States, and can be further anatomically classified as intrahepatic or extrahepatic based on their site of origin [1]. The incidence of CCA is projected to rise in the coming years, and prognosis is dismal for patients with unresectable or metastatic lesions [2,3]. Importantly, while local therapy options for intrahepatic and extrahepatic cholangiocarcinoma (ICC and ECC) can differ substantially, this anatomic distinction currently has little influence on decisions regarding upfront systemic therapy for advanced disease [4].

The landmark TOPAZ-1 trial established chemoimmunotherapy with durvalumab plus gemcitabine and cisplatin as the preferred first-line treatment regimen for advanced biliary tract cancer [5]. In this phase III study, the addition of the immune checkpoint inhibitor durvalumab to the aforementioned established chemotherapy backbone significantly improved overall survival at 2 years to 24.9% versus 10.4% with chemotherapy alone. The phase III KEYNOTE-966 trial subsequently confirmed the benefit of checkpoint inhibitor therapy in this setting and established pembrolizumab as a viable alternative to durvalumab [6]. Following progression on these first-line regimens, National Comprehensive Cancer Network (NCCN) guidelines recommend treatment with FOLFOX on the basis of the phase III ABC-06 trial [4]. This study compared FOLFOX plus active symptom control to active symptom control alone in the second-line treatment of advanced biliary tract cancer and reported modest but statistically significant improvement in median overall survival (OS) in the FOLFOX arm (6.2 months vs. 5.3 months, adjusted hazard ratio (HR) 0.69, 95% CI 0.50–0.97, *p* = 0.031) [7].

The marginal improvement in outcomes observed in the ABC-06 trial highlights the limitations of current CCA therapies. This need for additional therapeutic options, particularly in the second line and beyond, has prompted oncologists to become increasingly reliant on agents targeted against actionable genomic alterations identified via next generation sequencing (NGS). This “personalized” approach to CCA treatment has yielded promising results, with up to 40% of patients harboring actionable alterations [8,9,10]. Isocitrate dehydrogenase (*IDH*) gene mutations are amongst the most frequently encountered genetic aberrations, and use of agents targeting mutant IDH (mIDH) proteins, particularly mIDH1, is becoming increasingly common [8,9,10,11]. This review aims to summarize the biology of mIDH CCA and the current clinical approach to this rare subset of patients. Special emphasis is placed on emerging therapeutic strategies in preclinical and early phase clinical studies.

## 2. Isocitrate Dehydrogenase

IDH enzymes catalyze the oxidative decarboxylation of isocitrate to alpha-ketoglutarate (αKG) via a two-step process in which oxalosuccinate transiently forms as an intermediate [12]. The IDH protein family is composed of IDH1, present in the cytosol and peroxisomes, as well as IDH2 and IDH3, which both localize to the mitochondrial matrix [13]. IDH3 is structurally and functionally distinct from IDH1 and IDH2 (IDH1/2) in that it is a heterotetramer which catalyzes the irreversible generation of αKG and NADH in a tightly regulated step of the TCA cycle vital for cellular respiration [14]. In contrast, IDH1/2 are homodimers which catalyze a similar but reversible reaction in which αKG is produced alongside NADPH [13]. These two IDH isoforms exert a more pleiotropic influence on cellular signaling through regulation of intracellular αKG and NADPH levels.

αKG impacts gene expression through several epigenetic mechanisms which are mediated by αKG-dependent dioxygenases. Jumonji (Jmj) catalytic domain containing N-methyl-lysine and N-methyl-arginine demethylases represent one such group of oxygenases [15]. The ten-eleven translocation methylcytosine dioxygenase (TET) enzymes are another family of epigenetic regulators which demethylate cytosine nucleotides in DNA [16,17]. αKG is a required substrate in the demethylation reaction catalyzed by these enzymes, and thus fluctuation in its intracellular concentration can profoundly impact histone and DNA methylation and consequently gene transcription [18,19,20]. αKG-dependent dioxygenases also catalyze hydroxylation of several metabolic enzymes, such as hypoxia-inducible factor-1a (HIF-1α) [21,22].

In addition, IDH1/2 regulate cellular signaling through modulation of non-mitochondrial and mitochondrial NADPH pools [13]. NADPH acts as an intracellular reducing equivalent and required cofactor for enzymatic reactions involved in lipid biosynthesis, amino acid catabolism, and oxidative stress response [23,24,25,26]. Accordingly, IDH1 deficient mice demonstrate reduced gluconeogenesis, increased production of reactive oxygen species, and alterations in lipid metabolism and adipose distribution [25,27,28]. Likewise, IDH2 deficient animal models demonstrate severe mitochondrial dysfunction due to accumulation of oxidative damage in the absence of NADPH-dependent antioxidant regeneration [29].

## 3. Pathogenesis of IDH Mutant Cholangiocarcinoma

### 3.1. Mutant IDH and Oncometabolite R2HG

*IDH* mutations are relatively common in ICC and occur in defined hotspot regions: R132 for *IDH1* and R172 for *IDH2* [30]. These neomorphic “gain-of-function” mutations grant IDH1/2 the ability to reduce αKG into the R enantiomer of hydroxyglutarate (R2HG) at the expense of NADPH [31]. Through a concurrent loss in their affinity for isocitrate, mIDH proteins experience a 1000-fold reduction in their conventional αKG producing activity resulting in net consumption of intracellular αKG [31,32].

R2HG is consumed by few enzymatic reactions under physiologic conditions and accumulates to high concentrations (~5–30 mmol/L) in mIDH cancer cells [31,33,34,35]. As a small molecule that readily diffuses across cell membranes, R2HG can be detected in both the tumors and serum of CCA patients [36,37]. In these patients, circulating R2HG correlates with tumor burden and can predict *IDH* mutation status with a sensitivity of 83% and a specificity of 90% [36]. More recent data suggest that the ratio of serum R2HG to L enantiomer of hydroxyglutarate (which is produced under physiologic conditions and unaffected by malignancy) may predict *IDH* mutation status with even greater sensitivity and specificity (90% and 96.8%, respectively) [37]. In addition, oral mIDH inhibitors have been noted to reduce serum R2HG in clinical trials [38,39].

These findings suggest that quantitative assessment of serum R2HG could aid in the diagnosis of *IDH1/2* mutations. However, tissue biopsy remains indispensable for diagnosis of CCA [4]. In addition, NGS is required for identification of other genomic alterations, thus limiting the utility of R2HG as a diagnostic circulating biomarker [4]. Serum R2HG may also hold potential as a biomarker of therapeutic response, but these findings require validation in CCA [40].

### 3.2. R2HG Drives Epigenetic Dysregulation and Oncogenesis

The mechanisms underlying mIDH1/2-mediated oncogenesis were first described in AML and glioma [21,41,42,43]. Early experiments in glioma cell lines demonstrated that expression of mIDH1/2 was associated with reduction of intracellular αKG and diminished activity of αKG-dependent dioxygenases [21]. Further studies revealed that mIDH1/2-derived R2HG can bind αKG-dependent dioxygenases in place of αKG to directly inhibit their catalytic activity [42,43,44]. Accordingly, the presence of *IDH1/2* mutations in AML cells was associated with inactivation of TET2 5-methlycytosine (5mC) hydroxylase and a corresponding global increase in DNA methylation [41]. mIDH1/2-derived R2HG was similarly noted to inhibit TET2 and histone demethylases in glioma, resulting in increased DNA and histone methylation [43]. In both cancer types this aberrant epigenetic signaling resulted in altered gene expression and impaired differentiation of hematopoietic stem and neural progenitor cells [41,42]. Inappropriate expansion of these stem and progenitor cell populations has been proposed as the inciting event in mIDH1/2-mediated glioma and leukemia genesis [45,46]. Epigenetic dysregulation within m*IDH* cancers may also drive malignant behavior via overexpression of oncogenes such as PDGFRA and silencing of tumor suppressors such as CDKN2A [47,48]. However, it is hypothesized that additional somatic gene alterations are ultimately required for overt carcinogenesis [49,50,51,52,53].

Wang and colleagues were the first to characterize DNA methylation patterns in m*IDH1/2* cholangiocarcinoma [54]. Their study directly compared 19 m*IDH1/2* ICC tumors with 17 grade-matched *IDH* wild-type tumors and observed that the former displayed higher levels of DNA and histone methylation by immunohistochemistry (IHC). DNA methylation occurs at areas of repeating cytosine and guanidine dinucleotides termed CpG sites, which frequently overlap gene promoters. Further analysis with a microarray-based platform confirmed that 5758 CpG sites associated with 2309 genes in m*IDH1/2* tumors were hypermethylated relative to wild-type controls. Similar findings were reported in subsequent work by Jusakul et al. and Goeppert et al., who made use of the same microarray-based techniques [55,56].

More recently, Liao et al. used whole genome bisulfite sequencing (WGBS) to characterize a panel of 331 ICC samples [57]. Surprisingly, m*IDH* and wild-type tumors exhibited comparable levels of DNA methylation, and both were hypomethylated relative to non-malignant cholangiocytes. These discordant results compared to prior studies are likely the result of technical differences between WGBS and microarray-based techniques. Specifically, WBGS is capable of characterizing ~20 million CpG sites per sample, whereas previous microarray-based techniques were often limited to ~10,000 CpG sites [57]. These data suggest that m*IDH1/2* CCAs are not defined by a global increase in DNA methylation, but instead by a distinct pattern of DNA methylation at specific genomic sites.

As in AML and glioma, DNA hypermethylation in m*IDH1/2* CCA is hypothesized to result in oncogenic dysregulation of gene transcription. Epigenetic silencing of hepatocyte nuclear factor 4 alpha (*HNF-4α*) may be of particular importance [53]. The reduction in HNF-4α impairs hepatocyte differentiation and leads to accumulation of liver progenitor cells [53]. Importantly, these findings were replicated by exposure to exogenous R2HG and reversed by treatment with targeted mIDH inhibition or overexpression of HNF-4α [53]. This model of CCA genesis is supported by a liver progenitor cell-associated gene signature in m*IDH* tumors which is notably absent in *IDH* wild-type tumors [30,58].

**Figure 1:**
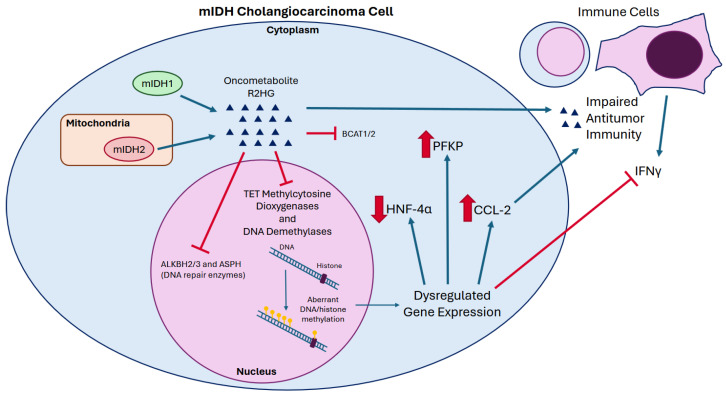
Pathogenesis of mutant IDH cholangiocarcinoma. Cytosolic mutant IDH1 and mitochondrial mutant IDH2 produce the oncometabolite R2HG which inhibits αKG dependent dioxygenase enzymes within the nucleus. This results in aberrant DNA and histone methylation and dysregulated gene transcription, including pathogenic silencing of the transcription factor HNF-4α, as well as overexpression of glycolytic enzyme PFKP and chemokine CCL-2. R2HG also directly inhibits the ALKBH2/3 and ASPH DNA repair enzymes, as well as the metabolic enzymes BCAT1/2. R2HG can diffuse into the tumor microenvironment where it directly inhibits antitumor immunity alongside secreted CCL-2. R2HG mediated epigenetic changes also blunt transcriptional response to IFNγ secreted by neighboring immune cells, further impairing antitumor immunity.

mIDH1/2 mediated epigenetic alterations also induce tumorigenic expression of the glycolytic enzyme phosphofructokinase-1 (*PFKP*). In an organoid model of intrahepatic biliary cells, R2HG mediated alterations in histone methylation upregulated expression of the platelet isoform of PFKP and stimulated aerobic glycolysis [59]. This enhanced organoid formation, augmented their survival in the face of metabolic stress, and decreased their reliance on exogenous growth factors in vitro [59]. Additionally, surgical m*IDH1/2* ICC specimens showed increased expression of PFKP by IHC relative to *IDH* wild-type samples [59]. R2HG also directly inhibits *ALKBH2/3* and *ASPH*, enzymes involved in repair of damaged DNA which may promote carcinogenesis [60,61]. In addition, R2HG inhibits branched chain aminotransferase (*BCAT*) 1 and 2, which are responsible for catabolism of branched chain amino acids (BCAAs) [62]. Of note, accumulation of the BCAAs valine, leucine, and isoleucine has been observed in ICC and implicated in proliferation of other solid cancers [63,64]. The diverse mechanisms by which mIDH1/2 are theorized to promote oncogenesis are summarized in Figure 1.

Accumulation of additional oncogenic mutations is presumably necessary to trigger transformation into overt ICC [53]. Unlike glioma, m*IDH1/2* are not strongly associated with *TP53* mutations in CCA [51,54]. m*IDH* ICCs also generally lack alterations in the mitogen-activated kinase pathway and *FGFR2* frequently observed in wild-type CCAs [9,54,57,65,66,67,68]. Recurrent co-alterations are instead observed in *ARID1A*, *PBRM1*, and *BAP1* [65,66,68], genes implicated in chromatin remodeling [69,70,71].

### 3.3. R2HG and the Immune Microenvironment

A comprehensive immunogenomic analysis of 33 tumor types revealed that m*IDH*-associated bulk transcriptomic signatures are indicative of reduced B-, natural-killer, and T-cell infiltration [72]. R2HG exerts a paracrine influence on intratumoral CD8+ T cells that suppresses their cytotoxic effector function [35]. R2HG also silenced expression of IFNγ dependent genes in a m*IDH1*-driven mouse model of CCA, thereby impairing the recruitment and activation of circulating immune cells [73]. More recently, Zabransky et al. demonstrated that m*IDH1* CCAs secrete CCL-2 which promotes accumulation of pro-tumorigenic M2 macrophages [74]. However, attempts to characterize an association between m*IDH1/2* and the immune microenvironment in clinical CCA samples have yielded inconsistent results [67,68,75,76]. mIDH1 inhibition appears to promote anti-tumor immunity and also sensitizes these tumors to checkpoint inhibitor therapy outlining a potential role for immunotherapy in this patient population [73,77,78].

## 4. Clinical Characteristics of mIDH Cholangiocarcinoma

*IDH1* and *IDH2* mutations are amongst the most frequently encountered genetic alterations in ICC, occurring in 10–20% and 2–5% of cases, respectively [4,8,9,79,80]. Their combined prevalence is far lower in ECC (less than 5% of cases), and no cases of m*IDH1/2* gallbladder cancer have been reported in the literature [8,9,79,80]. As is to be expected of a highly conserved component of the TCA cycle, mutations in *IDH3* are exceedingly rare in CCA and other malignancies [10,81]. *IDH* mutations occur more frequently in Europe and North America compared to Asia [82]. This geographic variation has been attributed to the increased pervasiveness of infectious causes of CCA in Asia such as hepatitis B, hepatitis C and liver-fluke, the presence of which is known to be inversely correlated with m*IDH* [83,84].

ICC can be histologically classified into small duct and large duct subtypes [85]. ICCs with m*IDH1/2* almost universally exhibit small duct morphology [86,87]. These tumors also demonstrate several unique pathologic features including a plump cuboidal/polygonal shape and increased geographic-type intratumoral fibrosis [86]. Expression of albumin mRNA by in situ hybridization is not associated with *IDH* mutation status [86]. Several studies have described m*IDH1/2* tumors as morepoorly-differentiated, consistent with their proposed origin as aberrant hepatic progenitor cells [79,88]. However, this has not been universally observed, with other studies reporting an association with moderate and well-differentiated tumors [87].

Early studies from China suggested that m*IDH1/2* ICC patients exhibited significantly prolonged OS and disease-free survival following surgical resection relative to IDH wild-type patients [54,87,89]. However, subsequent studies across a diverse range of Asian and non-Asian populations demonstrated no significant relationship between m*IDH* and OS [57,65,67,68,79,82,90,91]. These studies also reported inconsistent association between m*IDH1/2* and other demographic or pathologic variables such as age at diagnosis, stage at diagnosis, sex, venous invasion, perineural invasion, and pattern of metastasis. The inconsistencies may reflect underlying experimental bias, heterogeneity in geographic study location, the various clinic stages examined, and insufficient sample sizes.

NCCN guidelines recommend comprehensive molecular profiling of all metastatic and unresectable CCAs to detect potential *IDH1* gene alterations [4]. *IDH1* genotyping is frequently accomplished via IHC in other cancers such as glioma, but NGS is the preferred method in CCA as it enables simultaneous detection of actionable alterations in *FGFR2*, *NTRK*, *RET*, and *BRAF* [4,92]. *IDH2* mutation testing in CCA is not currently recommended by the NCCN guidelines, but the gene is frequently included in commercially available NGS panels [4]. At this time, use of mIDH1 targeted therapy is only recommended after progression on first line chemotherapy or chemoimmunotherapy [4]. *mIDH1* does not predict response to upfront chemotherapy [93], but preclinical data suggest mIDH1 may alter response to first-line immunotherapy [73,77,78].

## 5. mIDH Directed Therapies

### 5.1. Ivosidenib

Pharmacologic inhibition of mIDH1 reduced R2HG production and promoted differentiation of m*IDH1* mouse hepatoblasts harboring R132C or R132H polymorphisms in vitro [53]. This provided preclinical rationale to investigate ivosidenib, an orally bioavailable mIDH1 inhibitor, in CCA. Phase I/II studies revealed no unexpected toxicity at the 500 mg ivosidenib dose [94], and prompted the phase III randomized, double-blinded, multicenter, ClarIDHy trial [38,95,96]. In this study, 185 patients with m*IDH1* advanced CCA that had progressed on up to 2 prior systemic therapies were randomly assigned to either ivosidenib or placebo. Ivosidenib resulted in a significant improvement in median PFS compared to placebo (2.7 versus 1.4 months, HR 0.37, *p* < 0.0001) [95]. When adjusted for crossover, median OS was also higher in the ivosidenib arm (10.3 versus 5.1 months, HR 0.49, *p* < 0.001) [96]. Clinical benefit was largely derived from a maintenance of stable disease, as ivosidenib produced an objective response in only 2% of patients [95]. This drug was tolerated well, with only 2% of patients experiencing severe treatment-related adverse events, and only 6% of patients requiring treatment discontinuation due to treatment-emergent adverse events [96]. Patients receiving ivosidenib were also less likely to experience a significant reduction in quality of life compared to placebo, further supporting its tolerability [96]. These findings led to FDA approval of ivosidenib for adult patients with previously treated, locally advanced, or metastatic CCA with m*IDH1* [97]. Subsequent real-world data have validated its benefit with reported median PFS of 4.4 months and median OS of 15 months [98].

The molecular mechanisms which drive resistance, both innate and acquired, to ivosidenib are an ongoing area of research. Clinical studies have reported that baseline co-mutations in *PBRM1*, *ARID1A*, *PIK3CA*, *KRAS*, and *BAP1* are not associated with treatment response [94,96]. Based on gene expression profiling of 30 paired pre- and post-treatment tumor samples, mTORC1 and PI3K/AKT upregulation was associated with innate resistance to ivosidenib [77].

Secondary *IDH* mutations, IDH isoform switching, and alternative mutations in cellular differentiation pathways (e.g., *TET2* and *RUNX1*) are known mechanisms of acquired resistance to ivosidenib in AML [99,100,101]. In a small study, 6 of 37 patients treated with ivosidenib developed new oncogenic mutations in *IDH1* (R132C to R132F), *IDH2* (R172V), *TP53*, *ARID1A*, *POLE*, *PIK3R1*, and *TBX3* [94]. Other studies have also reported acquired IDH1/2 resistance mutations and the persistent dependence on R2HG [102]. Further research in a larger cohort is necessary to understand the relative frequency and mechanisms of action of these alterations to prevent or treat resistant tumors.

### 5.2. Olutasidenib

Olutasidenib (FT-2102) is a highly potent, orally bioavailable mIDH1 inhibitor with greater CNS penetration than ivosedinib [103]. It was approved by the FDA for treatment of relapsed m*IDH1* AML and appears to show greater therapeutic efficacy than ivosidenib in cross trial comparisons [104]. Olutasidenib was subsequently investigated in a phase Ib/II trial of 26 patients with relapsed/refractory m*IDH1* ICC [105]. In this small heavily pre-treated population, no complete or partial responses were observed, and only 6/26 (23%) patients exhibited stable disease. These results, along with those of other experimental mIDH1/2 inhibitors, are summarized in Table 1. There are several ongoing trials examining this agent in leukemia and glioma, but no further studies in CCA are listed on www.clinicaltrials.gov (accessed on 2 January 2025).

### 5.3. IDH305

IDH305, another inhibitor of mIDH1 with enhanced CNS penetration, was investigated in AML, glioma, and other solid tumors [106,107]. Unfortunately, due to a narrow therapeutic window, the phase I trial in AML was discontinued [114]. No results from solid tumor patients have been reported.

### 5.4. TQB3454

TQB3545 is another novel mIDH inhibitor targeting various mutations in the IDH1 kinase domain [108]. In a phase I trial in a heavily pre-treated CCA patient population (47.2% progressed on second-line therapy) in China, this agent demonstrated an ORR of 9.1%, DCR of 66.7%, median PFS of 4.7 months, and median OS 16.1 months [108]. These numbers compare favorably to historical data with ivosidenib, but larger trials are required.

### 5.5. LY3410738

IDH isoform switching is a mechanism of acquired ivosidenib resistance [94,102]. This has generated interest in dual mIDH1/2 inhibitors such as LY3410738. In a phase I trial, CCA patients receiving LY3410738 as monotherapy demonstrated a DCR of 4.5% and median PFS of 3.5 months [115]. In a subsequent dose expansion cohort, 13 treatment naive patients received LY3410738 in combination with cisplatin and gemcitabine [115]. This cohort demonstrated an ORR of 46% and a 6-month PFS rate of 83.3%. Additional research is necessary to confirm the therapeutic benefit of LY3410738, especially in combination with chemotherapy in the first-line setting.

### 5.6. HMPL-306

Phase I evaluation of another dual IDH1/2 inhibitor, HMPL-306, is also ongoing [116]. Results are awaited but, in preclinical studies in mIDH1/2 tumor xenograft mouse models, the drug markedly decreased 2-HG levels in plasma and tumor tissues [117]. Furthermore, this inhibition was reported to be more potent and durable than either ivosidenib or enasidenib.

## 6. Other Novel Therapeutic Strategies

The modest clinical benefit from ivosidenib is possibly due to insufficient suppression of R2HG [38]. Unfortunately, higher doses of ivosidenib do not result in further suppression of R2HG even though they are well tolerated [39,94]. As reviewed above, novel mIDH1/2 inhibitors exhibit comparable efficacy to ivosidenib in early phase trials, suggesting these agents may not dramatically improve patient outcomes as monotherapy (Table 1). *IDH1/2* alterations are “truncal” and represented in nearly all subclones within affected ICC tumors, validating the therapeutic utility of their inhibition [76,118]. However, secondary mutations in m*IDH1/2* tumors have prognostic significance and thus may impact response to therapy [119]. This raises concerns that genetic heterogeneity present in ICC tumors could serve as a nidus for acquired resistance [76,118]. Several novel therapeutic strategies and/or combinations are currently being explored as a means to overcome these potential therapeutic limitations. In addition, several potential targets other than mIDH1/2 are also under investigation in m*IDH* CCA.

### 6.1. mIDH Inhibitors and Cytotoxic Chemotherapy

Initial preclinical data in mIDH1 glioma and colon cancer cell lines suggested that mIDH inhibition may actually increase resistance to cisplatin [120]. However, the combination of mIDH inhibition with intensive chemotherapy improved response rates in AML [121]. In addition, the novel mIDH inhibitor LY3410738 combined with cisplatin and gemcitabine demonstrated superior efficacy to cisplatin and gemcitabine alone in early phase trials when compared to historical data [115,122]. The safety and feasibility of combining the mIDH1 inhibitor ivosidenib with first-line cisplatin and gemcitabine chemotherapy, as well as the PDL-1 inhibitor durvalumab, is the subject of a planned phase I trial (NCT06501625).

### 6.2. mIDH Inhibitors and Immune Checkpoint Inhibitor Therapy

In a genetically engineered mouse model of m*IDH1*-driven CCA, pharmacologic inhibition of mIDH1 enhanced cytotoxic T cell recruitment into the tumor microenvironment [73]. The cytotoxic effector function of these cells was then further augmented by checkpoint inhibitor blockade. Further work in the same model system showed that mIDH1 inhibitors also enhance antitumor innate immunity [78]. In addition, patient tumors demonstrate higher PDL1 expression following mIDH1 inhibitor treatment [77]. Based on these data, a phase II study is examining the combination of ivosidenib and nivolumab in patients with advanced *IDH1* mutant solid tumors (NCT04056910). The results of this study are awaited. Another phase I/II trial evaluated the feasibility of ipilimumab and nivolumab alongside ivosidenib as second-line treatment of m*IDH1* CCA (NCT05921760). Unfortunately, the trial closed early due to unacceptable toxicity [123]. Lastly, a phase I trial is planned to examine the safety and benefit of adding ivosidenib to first-line cisplatin, gemcitabine, and durvalumab (NCT06501625). Altogether, these studies will hopefully provide much needed insight into the therapeutic potential of this combination.

### 6.3. Src Kinase Inhibition

Recent preclinical data demonstrated that m*IDH1/2* cancers are dependent on Src kinase signaling, rendering them sensitive to Src inhibition [109,124]. An unbiased phosphoproteomic screen revealed that Src modifies membrane-associated guanylate kinase, WW and PDZ domain containing 1 (MAGI1) in m*IDH1* CCA [109]. This abrogates its tumor suppressive function as a component of the MAGI1–protein phosphatase 2A (PP2A) complex, resulting in activation of p70 S6 kinase (S6K) and enhancement of ribosomal protein synthesis [109]. The tumor suppressive function of MAGI1-PP2A could be restored via Src inhibition with dasatinib or the S6K/AKT inhibitor M2698, suggesting these agents may have therapeutic value in mIDH CCA [109]. Based on these data, a phase II trial of dasatinib in m*IDH* CCA is ongoing (NCT02428855).

### 6.4. PARP Inhibition

Poly ADP ribose polymerase (PARP) inhibitors are a class of antineoplastic agents which promote double-stranded DNA breaks and cell death in cancer cells lacking effective homologous recombination repair (HRR) [110]. Sensitivity to PARP inhibitors is classically associated with deleterious mutations in DNA repair proteins such as *BRCA1*, *BRCA2*, or *PALB2* [110]. However, R2HG analogously inhibits αKG-dependent dioxygenases involved in HRR, theoretically sensitizing these cancers to PARP inhibition [125]. Olaparib, a PARP inhibitor, showed limited activity as monotherapy in m*IDH1/2* glioma and CCA as subsequent therapy [111,125,126]. Later studies examined the use of olaparib alongside PDL1 blockade on the basis of preclinical data which showed that both PARP inhibition and mIDH inhibition enhance response to checkpoint inhibitor therapy [112,125]. This combination of olaparib and PDL1 blockade failed to show significant benefit in m*IDH* CCA as subsequent-line therapy. However, there may be a role for PARP inhibitors in platinum-sensitive m*IDH* CCA as a maintenance therapy [112,125]. In addition, two ongoing trials (NCT04298021 and NCT03878095) are investigating the combination of PARP inhibitors with the novel ataxia telangiectasia and Rad3-related (ATR) inhibitor ceralasertib.

### 6.5. Chloroquine and Metformin

The metabolic changes induced by mIDH increases their reliance on the electron transport chain and glutamine [113,127]. Metformin, an inhibitor of the electron transport chain, has been observed to reduce proliferation of m*IDH* cancer cell lines [128]. In addition, chloroquine, which inhibits the glutamine dehydrogenase mediated production of αKG, has been shown to reduce the production of R2HG [129]. The rational combination of metformin and chloroquine was examined in a phase Ib clinical trial of 17 patients with previously treated m*IDH1* solid tumors, 12 of whom had CCA [130]. This regimen was well tolerated with no dose-limiting toxicities observed, but no clinical responses were observed [130]. The use of phenformin, a more lipophilic metformin alternative with potentially greater intratumoral bioavailability, has been proposed as a candidate for future trials [130].

### 6.6. Other Agents

Hypomethylating agents (HMAs) are a mainstay of therapy in AML and myelodysplastic syndrome [121]. The combination of HMA in addition to mIDH1/2 inhibitors is based on the rationale that mIDH1/2 cancers have aberrant DNA hypermethylation [66,121]. Ivosedinib in combination with the HMA azacitidine was initially investigated as induction therapy in a phase II trial in AML patients not fit for intensive chemotherapy [131]. This combination resulted in a 70% complete response rate, far exceeding the historical rate of 27.5% associated with azacitidine monotherapy [131]. The phase III AGILE trial subsequently confirmed the benefit of this therapeutic strategy in patients with newly diagnosed m*IDH1* AML ineligible for intensive induction chemotherapy [132]. The addition of ivosidenib to azacitidine tripled median OS (24.0 vs. 7.9 months, HR 0.44, *p* = 0.001) without a significant increase in toxicity [132]. Furthermore, early trials evaluating the combination of the mIDH2 inhibitor enasidenib with an HMA have yielded similar results [121]. Given these data, exploration of this therapeutic combination should be considered in m*IDH1* CCA and other solid tumors.

Other potential therapeutic strategies in m*IDH1* CCA include inhibition of CDK9 and NAMPT. *IDH* mutant cancer cells are highly dependent on the NAD+ salvage pathway to maintain intracellular NADH pools [133]. Nicotinamide phosphoribosyltransferase (NAMPT) is the rate limiting enzyme in this salvage pathway, and its targeted inhibition caused NAD+ depletion and cell death in m*IDH1* glioma cell lines [133]. It similarly inhibited tumorigenesis and prolonged survival in m*IDH1/2*-driven mouse xenograft models [133]. Clinical trials of NAMPT inhibitors have been marred by poor efficacy and substantial toxicity [134]. However, these studies were not specifically performed in m*IDH* malignancies, and researchers are optimistic that more recently designed NAMPT-directed therapies and combinations may yield improved results [134]. Lastly, high throughput drug screening revealed that m*IDH1* glioma cells may be susceptible to the CDK9 inhibitor zotiraciclib, possibly due to increased vulnerability to ETC dysfunction [113,135]. If deemed safe and effective in ongoing clinical trials in glioma, then its use in m*IDH1/2* CCA warrants consideration.

## 7. Conclusions

The targeted inhibition of driver alterations has revolutionized outcomes in specific subsets of some malignancies, such as oncogene-addicted non-small cell lung cancers. However, additional research is still necessary if similar success is to be achieved in m*IDH* CCA. This includes further investigation into pathogenesis, biomarkers of innate and acquired mIDH inhibitor resistance, serum R2HG as a response biomarker, and novel combinational strategies with or without direct mIDH inhibitors. The modest clinical benefit of ivosidenib also demands that the cost-effectiveness of any novel drug be carefully considered across different healthcare settings, including resource-limited environments [136,137,138]. Ultimately, integration of basic, translational, and clinical research will be required to dramatically improve therapies and advance outcomes for our patients with m*IDH* CCA.

## Figures and Tables

**Table 1:** Comparative Efficacy of mIDH1 and Dual mIDH1/2 Inhibitors in Chemotherapy Refractory Cholangiocarcinoma.

Agent	Trial	Phase (n *)	Median Prior Lines of Therapy (Range)	ORR (%)	DCR (%)	Other Efficacy Endpoints	Citation
mIDH1 Inhibitors							
Ivosidenib	NCT02073994	Phase I (73)	2 (1–5)	4 (5)	45 (62)	Median PFS 3.8 mo Median OS 13.8 mo	[106]
Ivosidenib	NCT02989857 (ClarIDHy)	Phase III (124)	1 (1–2)	3 (2)	66 (53)	Median PFS 2.7 mo Median OS 10.3 mo	[107,108]
Olutasidenib	NCT03684811	Phase Ib/II (26)	2 (1–10)	0 (0)	6 (23)	NR	[109]
IDH305	NCT02381886	Phase I (24)	NR	NR	NR	NR	[110]
TQB3454	NCT04481607	Phase I (33)	≥1 (range NR ^‡^)	3 (9.1)	22 (66.7)	Median PFS 4.7 mo Median OS 16.1 mo	[111]
Dual mIDH1/2 Inhibitors							
LY3410738	NCT04521686	Phase I (42)	2 (1–7)	2 (4)	23 (56)	Median PFS 3.5 mo	[112]
HMPL-306	NCT04762602	Phase 1 (ongoing)	NR	NR	NR	NR	[113]

* Sample size includes only patients with cholangiocarcinoma who received mIDH inhibitor therapy; ^‡^ 47.2% of patients progressed on second-line therapy; Abbrv: mIDH (mutant isocitrate dehydrogenase), PFS (progression free survival), OS (overall survival), NR (not reported), ORR (objective response rate), DCR (disease control rate), mo (months).
